# Clock-Modulating Activities of the Anti-Arrhythmic Drug Moricizine

**DOI:** 10.3390/clockssleep3030022

**Published:** 2021-06-22

**Authors:** Chorong Han, Marvin Wirianto, Eunju Kim, Mark J. Burish, Seung-Hee Yoo, Zheng Chen

**Affiliations:** 1 Department of Biochemistry and Molecular Biology, The University of Texas Health Science Center at Houston, 6431 Fannin St., Houston, TX 77030, USA; Chorong.Han@uth.tmc.edu (C.H.); Marvin.Wirianto@uth.tmc.edu (M.W.); Eunju.Kim@uth.tmc.edu (E.K.); Seung-Hee.Yoo@uth.tmc.edu (S.-H.Y.); 2 Department of Neurosurgery, University of Texas Health Science Center at Houston, Houston, TX 77030, USA; Mark.J.Burish@uth.tmc.edu

**Keywords:** moricizine, circadian clock, period length, sodium channel blocker, heart arrhythmia, chronotherapy

## Abstract

Dysregulated circadian functions contribute to various diseases, including cardiovascular disease. Much progress has been made on chronotherapeutic applications of drugs against cardiovascular disease (CVD); however, the direct effects of various medications on the circadian system are not well characterized. We previously conducted high-throughput chemical screening for clock modulators and identified an off-patent anti-arrhythmic drug, moricizine, as a clock-period lengthening compound. In Per2:LucSV reporter fibroblast cells, we showed that under both dexamethasone and forskolin synchronization, moricizine was able to increase the circadian period length, with greater effects seen with the former. Titration studies revealed a dose-dependent effect of moricizine to lengthen the period. In contrast, flecainide, another Class I anti-arrhythmic, showed no effects on circadian reporter rhythms. Real-time qPCR analysis in fibroblast cells treated with moricizine revealed significant circadian time- and/or treatment-dependent expression changes in core clock genes, consistent with the above period-lengthening effects. Several clock-controlled cardiac channel genes also displayed altered expression patterns. Using tissue explant culture, we showed that moricizine was able to significantly prolong the period length of circadian reporter rhythms in atrial ex vivo cultures. Using wild-type C57BL/6J mice, moricizine treatment was found to promote sleep, alter circadian gene expression in the heart, and show a slight trend of increasing free-running periods. Together, these observations demonstrate novel clock-modulating activities of moricizine, particularly the period-lengthening effects on cellular oscillators, which may have clinical relevance against heart diseases.

## 1. Introduction

The mammalian circadian clock is our internal biological timer and plays a fundamental role in safeguarding health throughout our lifespan [1]. In response to the daily cycles of environmental changes from Earth’s rotation, the clock anticipates and adapts by orchestrating cellular and physiological functions throughout our body. The functional component of the clock is the ubiquitous cell-autonomous oscillator, consisting of highly conserved, interlocked negative feedback loops containing positive (e.g., CLOCK/NPAS2, BMAL1, RORs) and negative (e.g., PERs, CRYs, REV-ERBs) constituents. These cellular oscillators govern clock-controlled gene (CCG) expression directly, by the aforementioned core components, or indirectly, via secondary regulatory factors [2]. Systemic profiling has revealed very modest overlap in CCGs (~10%) among different tissues [3,4], indicating local clocks function specifically to regulate tissue physiology. On the other hand, oscillators in different tissues are also coordinated by the master pacemaker located at the suprachiasmatic nuclei (SCN) in the hypothalamus [5]. Collectively, more than 85–90% of mammalian genes display oscillatory expression in at least one tissue [3,4]. Such a prevalent circadian gene regulation underlies the numerous physiological roles of the clock in various organ systems.

A close link between the clock and the cardiovascular system is well documented [6,7,8,9,10,11]. For example, circadian patterns of cardiovascular functions have been shown in heart rate, blood pressure, endothelial function, QT interval, vascular contractility, and cardiometabolism. In accordance, cardiovascular attacks are also time-of-the-day dependent, including morning peaks for ischemic strokes and sudden cardiac death (SCD) [10]. Consistent with epidemiological evidence that shift workers are at a greater risk for various metabolic and cardiovascular disorders [12,13], mice subjected to a jet-lag paradigm followed by occlusion/reperfusion were found to suffer increased stroke infarct volumes [14]. A causal role of the clock in cardiovascular functions is further evidenced by studies of genetic mutants in mice [7,15,16,17]. For example, it has been shown that whole-body disruption of the core clock genes (*Clock* and *Bmal1*) abolished blood pressure rhythms and altered functions of the sympathetic nervous system [15]. Based on this cumulative knowledge of circadian regulation of cardiovascular (dys)functions, chronotherapy, namely timed administration of medications and treatments, is an active area of research and application [8,10]. For example, many studies have demonstrated that bedtime administration of anti-hypertensive agents markedly reduces sleep-time blood pressure, especially in the high-risk non-dipper patients when compared with morning intake [10]. While these studies illustrate how circadian timing alters CVD drug efficacy, the reciprocal process, namely the effects of CVD drugs on the intrinsic circadian clock, remains poorly characterized.

One question of great interest is the circadian mechanisms in SCD, the topic of a recent workshop, “Understanding Circadian Mechanisms of Sudden Cardiac Death”, convened by the National Institutes of Health, USA https://www.nhlbi.nih.gov/events/2019/understanding-circadian-mechanisms-sudden-cardiac-death (accessed on 11 June 2021). Acquired or hereditary heart diseases are the leading cause of SCD, and the primary mechanism leading to SCD is cardiac arrhythmias, particularly ventricular arrhythmias [18,19,20]. In the heart, membrane depolarization (sodium and calcium channels) and repolarization (potassium channels) are tightly regulated to maintain normal action potential, and adverse events causing exaggerated depolarization or diminished repolarization can disrupt the balance, increasing the risk of arrhythmias and ultimately SCD [21]. While the circadian regulatory mechanisms remain to be further studied, several studies in rodents highlight a key role of the clock in channel gene regulation. In one study, the essential clock gene *Bmal1* was conditionally deleted in adult cardiomyocytes in mice [22], causing slower heart rates and a greater risk of arrhythmia in the mice (iCSΔBmal1). Screening of channel genes revealed that *Scn5a*, encoding the major cardiac voltage-gated sodium channel Na_V_1.5, is a clock-controlled gene. Its oscillatory amplitude and expression levels were considerably reduced in iCSΔBmal1 mice, leading to attenuated Na+ current in ventricular myocytes [22]. Likewise, repolarization has also been shown to be subjected to circadian control [18,19]. For example, the transcription factor Krupple-like factor 15 (KLF15) is a CLOCK/BMAL1 target gene containing several E-box elements in its promoter region [23]. Importantly, KLF15 further directs the circadian expression of *KChIP2*, encoding an essential component of the voltage-gated potassium channel known to play an important role in cardiac repolarization [23]. Taken together, these studies provide initial mechanistic insights into the clock regulation of cardiac channel gene expression and arrhythmogenesis.

A number of anti-arrhythmic agents target cardiac ion channels. Moricizine, also known as ethmozin and moracizine, is an off-patent antiarrhythmic containing a phenothiazine backbone. Moricizine cannot be readily subclassified among Class I anti-arrhythmic drugs, as its clinical and electrophysiological effects overlap with drugs from several sub-classes [24,25]. Moricizine acts on the principle cardiac sodium channel Na_v_1.5 to reduce the fast inward sodium current for depolarization [24,25,26]. Moricizine also reduces the action potential duration (APD) to a greater degree than does the effective refractory period (EPR), resulting in a higher ERP/APD ratio [27]. As a result, membrane excitability is attenuated. Although moricizine has been shown to be effective in inhibiting spontaneous premature ventricular complexes and unsustained ventricular tachycardia, Cardiac Arrhythmia Suppression Trials (CAST and CAST-II) revealed an acute increase of mortality in the initial two-week treatment period and no improvement in survival during long-term follow-up [25]. Interestingly, the CAST trial showed an altered circadian distribution of SCD events in patients treated with moricizine and two other related drugs [28], suggesting an interaction with the circadian timing system.

The clock is a highly dynamic system amenable to modulation via a diverse array of cellular pathways [29,30]. Many studies, particularly those on high-throughput chemical screens, have provided evidence that various drugs are able to target circadian oscillators and physiological rhythms in vitro and in vivo, altering circadian period, phase, and amplitude via diverse mechanisms [31,32,33,34,35,36]. Previously, we performed several studies to discover and characterize chemical compounds, including drugs, that manipulate circadian oscillators [37,38,39]. In an initial chemical screen using the Per2::LucSV reporter fibroblast cells [37], we identified moricizine as a clock-modulatory drug that alters the reporter circadian rhythms. Here, we report the characterization of clock-modifying activities of moricizine in vitro and in vivo, and discuss its potential implication.

## 2. Results

### 2.1. Clock-Altering Activities of Moricizine

**Figure 1:**
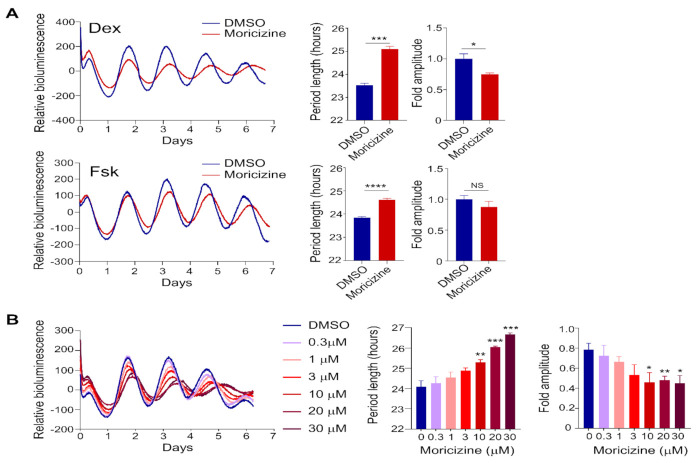
Moricizine lengthens the circadian period of mouse Per2::LucSV reporter fibroblast cells. (**A**) Representative PER2::LUC bioluminescence recording of Per2::LucSV fibroblast cells. Cells were synchronized by Dex (200 nM, *n* = 3) or Fsk (1 μM, *n* = 8). Average circadian period lengths and amplitudes show a period-lengthening effect with moricizine (10 μM), compared to controls. (**B**) Representative PER2::LUC bioluminescence recording, average circadian period lengths and amplitudes of Per2::LucSV fibroblast cells treated with increasing concentrations of moricizine (*n* = 4 for each concentration). Data are presented as mean ± SEM. *T*-test shows significant statistical differences between DMSO and moricizine. (*, *p* < 0.05; **, *p* < 0.01; ***, *p* < 0.001; ****, *p* < 0.0001).

Previously, we conducted high-throughput chemical screens and identified synthetic compounds and natural products that alter circadian reporter rhythms and physiological outputs [37,38]. As part of the initial screen [37], we also found an off-patent anti-arrhythmic drug called moricizine that lengthened the circadian reporter rhythms (Figure S1A,B). To further investigate this potential clock-modulating effect, we grew Per2::LucSV reporter cells in 35 mm dishes to confluency, and synchronized by dexamethasone (Dex, 200 nM) or forskolin (Fsk, 1 µM), two commonly used agents for cellular clock synchronization [37,39,40]. As shown in Figure 1A, moricizine was found to lengthen reporter rhythms under both conditions (Dex: DMSO, 23.5 ± 0.1 h vs. moricizine, 25.1 ± 0.1 h; Fsk: DMSO, 23.8 ± 0.05 h vs. moricizine, 24.7 ± 0.05 h). The period-lengthening effect was greater with Dex. Moreover, amplitude was also reduced by moricizine when cells were treated with Dex; in contrast, this effect was not significant with 1 µM Fsk treatment. Interestingly, however, when cells were pre-treated with a higher dose of Fsk (5 µM), moricizine displayed both period-lengthening and amplitude-reducing effects (Figure S1C).

We next conducted dose-response tests following Dex treatment. As shown in Figure 1B, cells were treated with DMSO (vehicle) or moricizine at concentrations ranging between 0.3 to 30 µM. We observed progressive effects of period lengthening and amplitude reduction as a function of the moricizine dose, first becoming significant at 10 µM and peaking at 30 µM. Together, these results establish novel clock-altering activities of moricizine.

### 2.2. Flecainide Does Not Affect Circadian Reporter Rhythms

**Figure 2:**
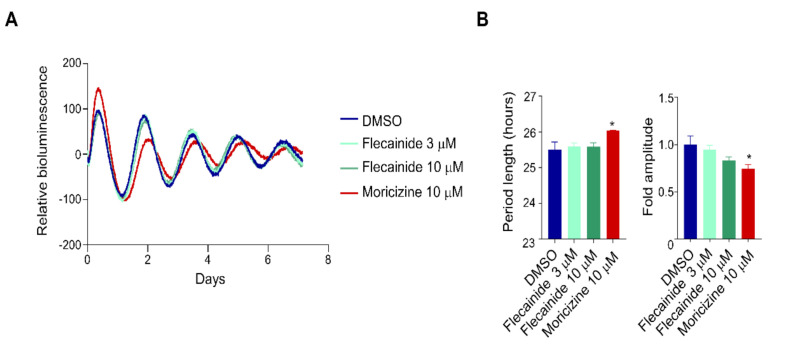
Flecainide does not affect circadian reporter rhythms. (**A**) Representative PER2::LUC bioluminescence recording of Per2::LucSV fibroblast cells treated with Figure 3. or 10 μM) or moricizine (10 μM). (**B**) Average circadian period lengths and amplitudes are shown (*n* = 3–4 for each concentration). Data are presented as mean ± SEM. *T*-test shows significant statistical differences between DMSO and flecainide or moricizine. (*, *p* < 0.05).

We next investigated the effect of flecainide, a Class Ic anti-arrhythmic drug that shares functional features with moricizine in reducing the duration of action potential and was investigated together with moricizine in the CAST trial [24,28]. Unlike moricizine, flecainide showed no effects on the PER2::LUC reporter rhythm at 3 µM, 10 µM (Figure 2), or 30 µM (Figure S2). This result underscores a specific clock-modulatory effect of moricizine.

### 2.3. Moricizine Regulates Circadian and Cardiac Channel Gene Expression

**Figure 3:**
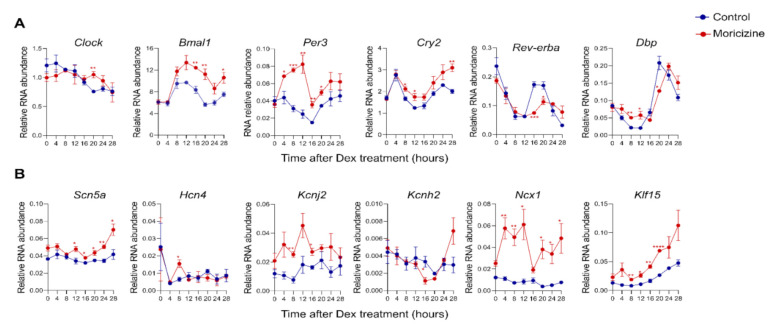
Real-time qPCR analysis of circadian and cardiac gene expression. Circadian ((**A**); *Clock*, *Bmal1*, *Per3*, *Cry2*, *Rev-erba*, and *Dbp*) and cardiac ((**B**); *Scn5a, Hcn4, Kcnj2, Kcnh2, Ncx1,* and *Klf15*) gene expression in Per2::lucSV reporter cells quantified by qPCR for control (blue) and moricizine 20 µM (red). Data are shown as mean ± SEM every 4 h for 28 h (*n* = 3). *T*-test shows the significant statistical differences between DMSO and moricizine at the indicated time points. Two-way ANOVA analysis shows significant differences between DMSO and moricizine for *Bmal1*, *Cry2*, *Per1*, *Per3*, *Scn5a*, *Kcnj2*, *Ncx1*, and *Klf15*. (*, *p* < 0.05; **, *p* < 0.01; ***, *p* < 0.001; ****, *p* < 0.0001).

To further investigate how moricizine alters the core oscillator, we collected moricizine-treated Per2::LucSV reporter cells over a complete circadian cycle and performed real-time qPCR analysis to examine the effects of moricizine on clock gene expression. We observed significant circadian time- and/or treatment-dependent changes in most core clock genes, indicating a strong modulatory effect of moricizine on the core oscillator (Figure 3A and Figure S3A). Of note, several circadian genes, including the core clock genes *Bmal1*, *Cry2*, *Per3,* and *Rev-erba,* as well as the clock output gene *Dbp*, displayed altered rhythmic expression patterns that are consistent with a period-lengthening effect of moricizine.

Cardiac arrhythmias are closely linked with ion channel expression and function, including that of the moricizine target Na_v_1.5, encoded by *Scn5a* [22]. Furthermore, *Scn5a* and several other key ion channel or regulatory genes are under circadian control [19]. Therefore, we next investigated how moricizine modulates expression of these ion channel and regulatory genes (Figure 3B and Figure S3B). We observed significant expression changes in these channel genes over the circadian time course, most notably *Scn5a*, *Kcnj2*, *Ncx1,* and *Klf15*.

### 2.4. Moricizine Affects Tissue Clocks

**Figure 4:**
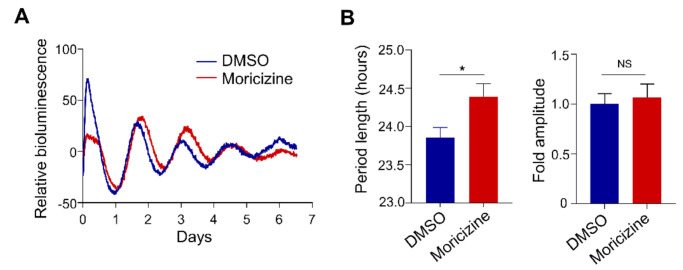
Moricizine lengthens the circadian period in atrial tissues ex vivo. (**A**) Representative PER2::LUC bioluminescence recordings of atrial tissues from Per2::Luc mice (blue traces for DMSO, red traces for 30 µM moricizine-treated atrial tissues, *N* = 10-11 for each group). (**B**) Period comparisons and relative fold amplitudes of DMSO or moricizine treated atrial tissues were calculated by the LM fit (damped sin) method. Data are presented as mean ± SEM. *T*-test shows significant statistical differences between DMSO and flecainide or moricizine. (*, *p* < 0.05).

To begin to explore the clock-modulatory activities of moricizine in a more physiological setting, we performed tissue explant experiments using Per2::Luc mice as previously described [39,41]. We specifically focused on cardiac tissue explants. Repeated tries did not yield consistent circadian recording using ventricle tissues, similar to previous observations [42]. Therefore, we focused on atrial tissues. As shown in Figure 4, in accordance with fibroblast cell culture results shown in Figure 1, moricizine was able to significantly lengthen the circadian period in atrial explants (23.9 ± 0.2 h vs. 24.6 ± 0.2 h), while the amplitude effect was not significant.

### 2.5. In Vivo Effects of Moricizine on Sleep and Circadian Activity

**Figure 5:**
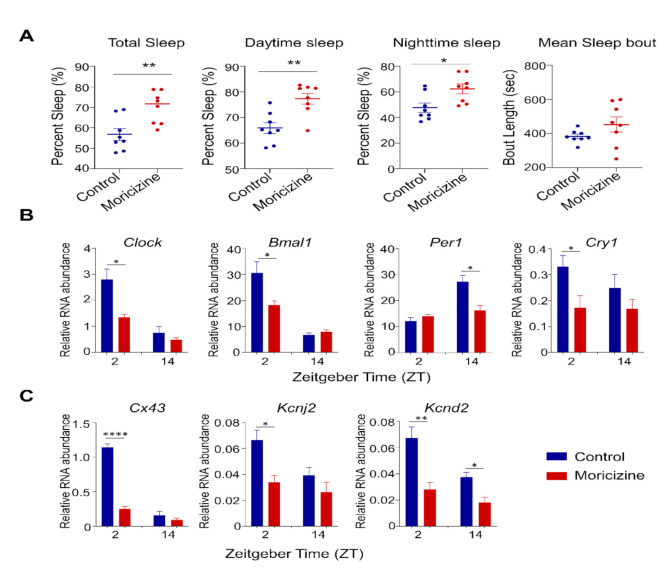
Moricizine effects on sleep and heart gene expression in C57BL6/J mice. (**A**) Total percent sleep time, daytime sleep time, nighttime sleep time, and mean sleep bout length of wild-type C57BL/6J mice with vehicle (PBS, blue, *n* = 8) or moricizine (1.25 mg per mouse, red, *n* = 8). Real-time qPCR analysis of clock genes ((**B**); *Clock, Bmal1, Per1,* and *Cry1*) and cardiac channel ((**C**); *Cx43, Kcnj2*, and *Kcnd2*) in heart tissues of control (blue bar, *n* = 4 for ZT 2 and ZT14) and moricizine treated mice (red bar, *n* = 4 for ZT2 and ZT14). Data are presented as mean ± SEM. *T*-test shows significant statistical differences between control and moricizine treated groups. (*, *p* < 0.05; **, *p* < 0.01; ****, *p* < 0.0001).

We next investigated in vivo effects of moricizine in WT C57BL/6J mice. Based on the daily dose in human, systemic bioavailability, and human–mouse equivalency (see M&M), we performed IP injection of moricizine at a daily dose of ~50 mg/kg for mouse in vivo studies. For sleep studies, we performed daily IP injection for 5 days at ZT2 (2 h after light on, inactive phase). We found that moricizine increased the amount of sleep, including total, daytime, and nighttime amounts (Figure 5A). Measurement of hourly distribution revealed distinct temporal patterns of sleep, with significant increases in early nighttime sleep in moricizine-treated mice (Figure S4A). Interestingly, sleep–wake decision statistical analysis [43] showed a significantly lower sleep–wake transition threshold in the moricizine group (Figure S4B, 0.157 vs. 0.652), suggesting that moricizine-treated mice are more prone to transition between sleep and wakefulness. On the other hand, mean sleep bout was not significantly altered, although moricizine-treated mice exhibited a greater variation in sleep bout length (Figure 5A and Figure S5).

We next collected heart tissues at ZT2 and ZT14, and performed real-time qPCR analysis to investigate whether moricizine modulates circadian gene expression in the heart (Figure 5B,C and Figure S6). Several clock and clock-controlled genes were found to exhibit altered expression in a circadian time-specific manner, particularly ZT2. For example, both *Clock* and *Bmal1* expression levels were reduced at ZT2, as well as *Cry1*, *Cx43*, and *Kcnd2*. However, *Per1* expression was changed only at ZT14.

**Figure 6:**
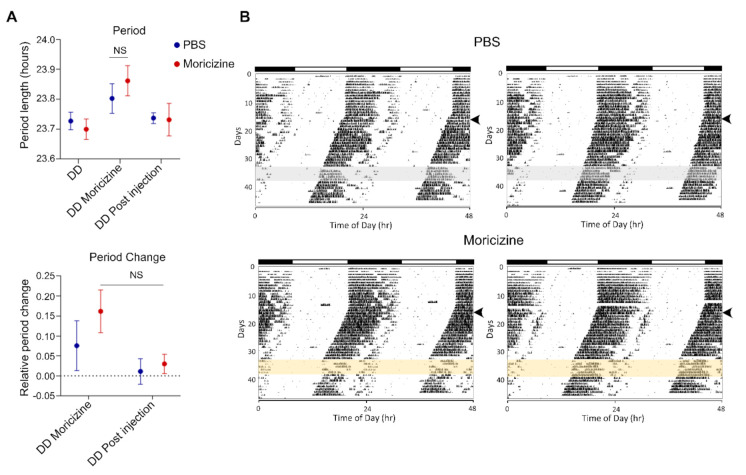
Moricizine shows trends of period lengthening and phase shift in mice. (**A**) Free-running period and relative period change of C57BL6/J male under DD (constant darkness) before IP injection, DD Moricizine for during IP injection, and DD Post injection for post IP injection. (**B**) Representative actograms are shown for C57BL6/J male mice. Arrows indicate the LD (light:dark) to DD (dark:dark) transition. PBS (*N* = 7) or moricizine (1.25 mg per mouse, *N* = 6) was injected by IP during the interval indicated by gray or yellow shading, respectively, on the actogram.

Finally, to determine the effect of moricizine on mouse voluntary wheel-running behavior, WT C57B/6 mice were allowed to free-run in constant darkness before moricizine was IP injected at circadian time 11 (CT11), just before activity onset (CT12), for five days. As shown in Figure 6A (summary data) and Figure 6B (representative actograms), while the free-running periods were virtually identical before and after treatment (DD and DD Post injection, respectively), there was a slight trend, without reaching statistical significance, of period lengthening and phase shift during the treatment (DD moricizine). Finally, moricizine treatment also led to a marginal effect on circadian activity pattern (Figure S7A) and total activity (Figure S7B), although again not reaching statistical significance.

## 3. Discussion

In this work, we report novel clock-altering activities of the anti-arrhythmic drug moricizine. Using Per2::LucSV circadian reporter fibroblast cells, we observed dose-dependent period-lengthening effects of moricizine. Circadian amplitude was also reduced with Dex synchronization. Several core clock genes were found to display altered circadian expression patterns consistent with period lengthening by moricizine. In accordance with core clock modulation, expression of several clock-controlled ion channel genes was also changed in a circadian time-dependent manner. At the tissue level, atrial ex vivo explant cultures treated with moricizine also exhibited longer circadian periods. In vivo tests showed that a human-equivalent dose of moricizine led to pronounced increases in the amount of sleep and altered circadian gene expression in the heart. Circadian wheel-running assays revealed a slight trend of period lengthening in voluntary exercise behaviors. The reason for the small effect on wheel running behavior, known to be controlled by the SCN [5], could be that the SCN oscillators are more tightly coupled than peripheral counterparts and are thus resistant to genetic and chemical perturbations [37,38,44]. Together, our results reveal a role of moricizine in manipulating the cardiac and systemic clocks.

The detailed molecular mechanism underlying the clock-manipulating effects of moricizine remains to be further investigated. Although it is generally believed to inhibit the major sodium ion channel Na_v_1.5 encoded by the *Scn5a* gene, whether and how this activity may be linked to its circadian function remains unclear. *Scn5a* is itself a clock-controlled gene, harboring an E-box element recognized by the canonical circadian activator CLOCK/BMAL1 [22]. However, the reciprocal effect of *Scn5a* on the circadian system is unknown. Previously, a genome-wide siRNA screen was conducted in human osteosarcoma cell line U2OS to profile clock-modifying genes globally [45], and knockdown of *Scn5a* did not significantly alter circadian periodicity of the Bmal1-Luc reporter rhythm. One caveat, however, is that *Scn5a* expression is enriched in the heart, and at much lower levels in other tissues. Therefore, whether *Scn5a* may regulate circadian rhythms, or is involved in the observed moricizine effect of manipulating circadian rhythms, remains to be investigated. Alternatively, moricizine may impinge on a new and unknown target to exert its clock effects. As shown previously [31,32], small-molecule drugs can have promiscuous effects which influence circadian rhythms. Finally, the drug metabolism is known to be regulated by the clock [46,47,48], suggesting potentially reciprocal interactions between drugs and circadian rhythms. Regardless, future studies will be needed to investigate these possibilities and further characterize the role of moricizine in modulating the clock network.

The majority of top-selling drugs target proteins expressed from clock-controlled genes [3]. Several chemical screens have also revealed robust effects of a small number of drugs, notably kinase inhibitors, to alter circadian period, phase, and amplitude [31,32,34,36,49]. However, detailed characterization is lacking for most of these agents. We recently characterized the circadian effect of verapamil, a first-line medication for cluster headaches known to display a strong circadian pattern of attacks [39]. In circadian reporter cells and in wheel-running assays in vivo, we showed that verapamil shortens circadian periods. Interestingly, verapamil is a Class IV anti-arrhythmic and a L-type calcium channel inhibitor. The current study shows a distinct circadian period effect of the Class I drug moricizine, suggesting differential mechanisms by these anti-arrhythmic drugs to alter tissue-specific circadian timing. Previously, the CAST trial revealed that the circadian pattern of arrhythmia-related mortality in patients receiving the treatment (encainide, flecainide, or moricizine) was altered relative to the placebo group, showing a closely clustered, bimodal peak pattern [28]. It should be noted, however, that this clinical study did not specifically stratify patients receiving moricizine or other drugs, and our study shows that Flecainide, unlike moricizine, did not alter the clock. It remains to be further investigated whether the clock-altering activities of moricizine reported herein may contribute to the changes in circadian time of death in the CAST trial. Our in vivo studies also provide evidence that mice slept significantly more after receiving doses equivalent to daily intake by human patients. How these effects may relate to side effects of moricizine, such as dizziness, awaits further investigation.

Our study may have important therapeutic implications. Circadian rhythms are now appreciated as a fundamental mechanism for health and disease [1,3,50]; however, translation of this knowledge to medical advances is lagging. Given the established close links between the clock and the cardiovascular system, notable successes have been achieved in chronotherapy for CVD [8,10]. As mentioned above, bedtime dosing of antihypertensives has been widely adopted to normalize nighttime systolic blood pressure and blunt the morning peak of adverse events [51]. Interestingly, chronotherapy can also be applied to surgical intervention, as aortic valve replacement surgery performed in the afternoon led to significantly improved prognoses relative to morning procedures in a randomized clinical study [52]. On the other hand, rather than altering therapeutic timing, clock manipulation by pharmacological or behavioral interventions may also improve cardiovascular performance, as shown by preclinical studies using a small-molecule agonist for the core clock component REV-ERVs [53,54], or a time-restricted feeding paradigm [55,56]. Likewise, our previous work also showed that a clock-enhancing natural compound was able to prevent metabolic disease and promote healthy aging [38,57,58]. These proof-of-concept laboratory studies underscore a promising venue of therapeutic development by directly targeting the clock [33]. In designing clock-based therapies, it is important to consider the effects of the drugs on intrinsic physiology. As illustrated by our study, existing drugs may significantly modify the internal clock system, with beneficial or deleterious effects related to efficacy or toxicity, respectively.

In conclusion, we demonstrate here that moricizine, an off-patent Class I anti-arrhythmic, is able to modulate the circadian clock, particularly by lengthening the circadian period and altering circadian and cardiac gene expression and behaviors in vivo. Future studies should determine the cellular mechanism and examine how these circadian effects may play a role in its therapeutic efficacy and/or toxicity.

## 4. Materials and Methods

### 4.1. Animals

Animal husbandry and experiments were carried out according to IACUC guidelines and an animal protocol (AWC-20-0058) approved by the University of Texas Health Science Center at Houston (UTHSC-H).

### 4.2. Circadian Reporter Luminescence Monitoring

Real-time circadian bioluminescence monitoring was conducted by using adult mouse ear fibroblast cells isolated from Per2::LucSV knock-in mice by replacement of the 3′-UTR with an SV40 late poly(A) sequence as described [59]. Cells were grown to confluency on 35 mm dishes in Dulbecco’s Modified Eagle’s Medium (DMEM) medium supplemented with 10% Fetal Bovine Serum (FBS) and 1% penicillin/streptomycin, and subsequently synchronized with 200 nM dexamethasone (Dex) for 1.5 h or forskolin (Fsk, 1 or 5 µM) for 1 h [37,41,59]. Moricizine (Sigma–Aldrich or Santa Cruz) was then added at concentrations of 0 (vehicle control), 0.3, 1, 3, 10, 20, and 30 µM, or Flecainide (Sigma-Aldrich) at 3, 10, and 30 µM, along with luciferin-containing recording media. The dishes were then tightly sealed with vacuum grease and placed in a LumiCycle luminometer (Actimetrics) for continuous bioluminescence monitoring over six days. The data were detrended using a first-order polynomial, then best-fit to a sine wave estimated by a Levenberg–Marquardt algorithm for measurement of circadian parameters in the LumiCycle data analysis program (Actimetrics).

### 4.3. Real-Time RT-PCR Analysis

RNA extraction and real time RT-PCR analysis were carried out as previously described [60,61]. For real-time qPCR analysis of core clock genes in cultured cells, Per2::LucSV cells were synchronized with 200 nM Dex as above. Moricizine was then added at concentrations of 20 µM in DMEM containing 2% FBS. Cells were harvested every 4 h for 28 h (eight time points) and total RNA were extracted by using PureXtract RNAsol Reagent (GenDEPOT, Houston, TX, USA) as indicated by the manufacturer’s protocol. For heart tissue gene expression analysis, heart tissues were collected at the indicated times (ZT2 and ZT14) for homogenization and RNA extraction. Reverse transcription was performed by cDNA synthesis kit (GenDEPOT, Houston, TX, USA). All real-time RT-PCR reactions were performed with SYBR Green PCR Master Mix kits (GenDEPOT, Houston, TX, USA) on QuantStudio 7 Flex system (Applied Biosystems). Data were analyzed using Prism 8 software (GraphPad Software, Inc., San Diego, CA, USA). *Gapdh* were used as the housekeeping gene for controls. The primer sets used are shown in Table 1.

### 4.4. Atrium Ex Vivo Cultures for Real-Time Circadian Bioluminescence Monitoring

Ex vivo circadian bioluminescence measurement was performed as previously described (Yoo et al., 2004). Briefly, hearts were quickly removed and placed in warm PBS solution to remove blood. Left and right atrial tissues was manually dissected to small pieces of equivalent size (four pieces per atrium); both right and left atria were used. The dissected atrial tissues were cultured on Millicell culture membranes (PICMORG50, Millipore) in 35 mm dishes containing 2 mL DMEM media (Invitrogen) supplemented with 352.5 μg/mL sodium bicarbonate, 10 mM HEPES (Invitrogen), 2 mM l-Glutamine, 5% FBS, 25 units/mL penicillin, 25 μg/mL streptomycin (Invitrogen), and 0.1 mM luciferin (l-8240, Biosynth AG). DMSO or moricizine (30 µM) was added to the recording media. Bioluminescence was recorded continuously using the LumiCycle luminometer (Actimetrics). Data were analyzed using LumiCycle data analysis program (Actimetrics).

### 4.5. In Vivo Dose Consideration and Animals

The dose for in vivo experiments using mice was calculated as follows. In human patients, moricizine is prescribed for 200–300 mg, three times a day, corresponding to a total daily dose of 600–900 mg/day for an average body weight of 60 kg. To avoid circadian disturbance from repeated administration throughout the day, we decided to perform one-time daily IP injection at a dose equivalent to the lower human dose at 600 mg/day/person. Taking into account the human/mouse dosing ratio of 12.3 [62] and the oral bioavailability of ~38% [24], we estimated that for a young adult male mouse at 25 g, one-time daily IP injection would require 1.25 mg moricizine.

C57BL6/J mice (Jackson Laboratory) at nine weeks of age were transferred into individual cages equipped with running wheels. Mice were acclimated in a 12 h light/12 h dark cycle (LD, light levels 300 lux, room temperature and relative humidity were maintained at 22.6–24.1 °C and 38–42%, respectively) before experiments.

### 4.6. Piezo Sleep Recording

Acclimated mice were treated by daily IP injection (1.25 mg moricizine per mouse in 100 µL PBS, or 100 µL PBS alone for the control group) for five days. We chose to inject at ZT2 to minimize acute effects of light on at ZT0, also considering the relatively short half-life of moricizine [24,25]. Sleep/wake recording was performed with a noninvasive piezoelectric transducer sleep/wake recording system (Signal Solutions, Inc., Lexington, KY, USA) as previously described (Nohara et al., 2019). The initial 48 h acclimation period was followed by data recording for five days. Data were extracted and analyzed by using the Sleepstats software [43] (Signal solutions, Inc., Lexington, KY, USA).

### 4.7. Mouse Wheel-Running Behavioral Study

Acclimated mice were housed for two weeks in constant darkness (DD) to measure baseline free-running periods and to deduce average daily shift in activity onset. Once a circadian period was established in DD (the free-running period), we monitored the effects of moricizine on the free-running period by IP injection as above, followed by an additional seven-day period in DD. Activity data were recorded continuously by a PC system (Chronobiology Kit, Stanford Software Systems) and analyzed using CLOCKLAB software (Actimetrics). Free-running period was calculated using activity onset time with Chi-square periodogram (CLOCKLAB). Wheel-running activity level was quantified as a summation every 20 min for each mouse and averaged for entire experimental duration.

### 4.8. Statistical Analysis

Sample size was based on previous studies [39,58]. Data are presented as mean ± standard deviation (cell culture data) or mean ± standard error of the mean (wheel-running and sleep data). Statistical significance was determined by two-tailed Student’s *T*-test (Excel, Microsoft Office Professional Plus version 2016) and two-way ANOVA with Sidak’s multiple comparison test (GraphPad Prism version 8.20). *p* < 0.05 was considered statistically significant.

## Figures and Tables

**Table 1:** qPCR primer sequences.

Gene	Forward (5′-3′)	Reverse (5′-3′)
*Clock*	CCTTCAGCAGTCAGTCCATAAAC	AGACATCGCTGGCTGTGTTAA
*Bmal1*	CCACCTCAGAGCCATTGATACA	GAGCAGGTTTAGTTCCACTTTGTCT
*Per1*	CCCAGCTTTACCTGCAGAAG	ATGGTCGAA AGG AAGCCTCT
*Per2*	TGTGCGATGATGATTCGTGA	GGTGAAGGTACGTTTGGTTTGC
*Per3*	GTGATTGTTCACGCGTCT GT	CACTGCCATCTCGAGTTCAA
*Cry1*	TGA GGC AAG CAG ACT GAA TAT TG	CCT CTG TAC CGG GAA AGC TG
*Cry2*	CTG GCG AGA AGG TAG AGT GG	GACGCAGAATTA GCCTTTGC
*Rev-erba*	CATGGTGCTACTGTGTAAGGTGTGT	CACAGGCGTGCACTCCATAG
*Rev-erbb*	TGAACGCAGGAGGTGTGATTG	GAGGACTGGAAGCTATTCTCAGA
*Dbp*	CTGGCCCGAGTCTTTTTGC	CCAGGTCCACGTATTCCACG
*Scn5a*	GCAGAAGGTGAAGTTCGTGG	TGAAGACCAAGTTTCCGACC
*Hcn4*	CGACAGCGCATCCATGACTA	GCTGGAAGACCTCGAAACGC
*Kcnj2*	TGAAGTTGCCCTAACAAGCA	AAAGTAAGTATGACAAAGACGGAA
*Cacna1c*	CCCTTCTTGTGCTCTTCGTC	TATGCCCTCCTGGTTGTAGC
*Kcnh2*	GATCGCCTTCTACCGGAAA	CATTCTTCACGGGTACCACA
*Ncx1*	GAGGTTGGAGCAGTTGGAAG	ACCAGACGAAATCCCATTGA
*Klf15*	TGTGGGCCAGAAGTTTCC	AAGTGCATTTGTGCATTTTGAG
*Cx43*	GAGAGCCCGAACTCTCCTTT	TGGGCACCTCTCTTTCACTT
*KChIP2*	AACTATCCACGGTGTGCCAC	GGACATTCGTTCTTGAAGCCT
*Kcnd2*	GCAAGCGGAATGGGCTAC	TGGTTTTCTCCAGGCAGTG
*Gapdh*	CAAGGTCATCCATGACAACTTTG	GGCCATCCACAGTCTTCTGG

## Data Availability

All raw data are available from the corresponding author upon reasonable request.

## Supplementary Materials

The following are available online at https://www.mdpi.com/article/10.3390/clockssleep3030022/s1, SM contains 7 supplementary figures and corresponding figure legends.
