# Supplementary material for: Clock-Modulating Activities of the Anti-Arrhythmic Drug Moricizine
Source: Clocks Sleep. 2021 Jun 22;3(3):351–65. doi: 10.3390/clockssleep3030022 (PMC8293187; doi:10.3390/clockssleep3030022)
Supplement: Supplementary file 1 [file clockssleep-03-00022-s001.zip › clockssleep-1240397-supplementary.pdf]

Supplementary Materials

Han et al., “Clock-modulating activities of the anti-arrhythmic drug moricizine”

Containing 7 supplementary figures (Figure S1-S7) and supplementary figure legends.

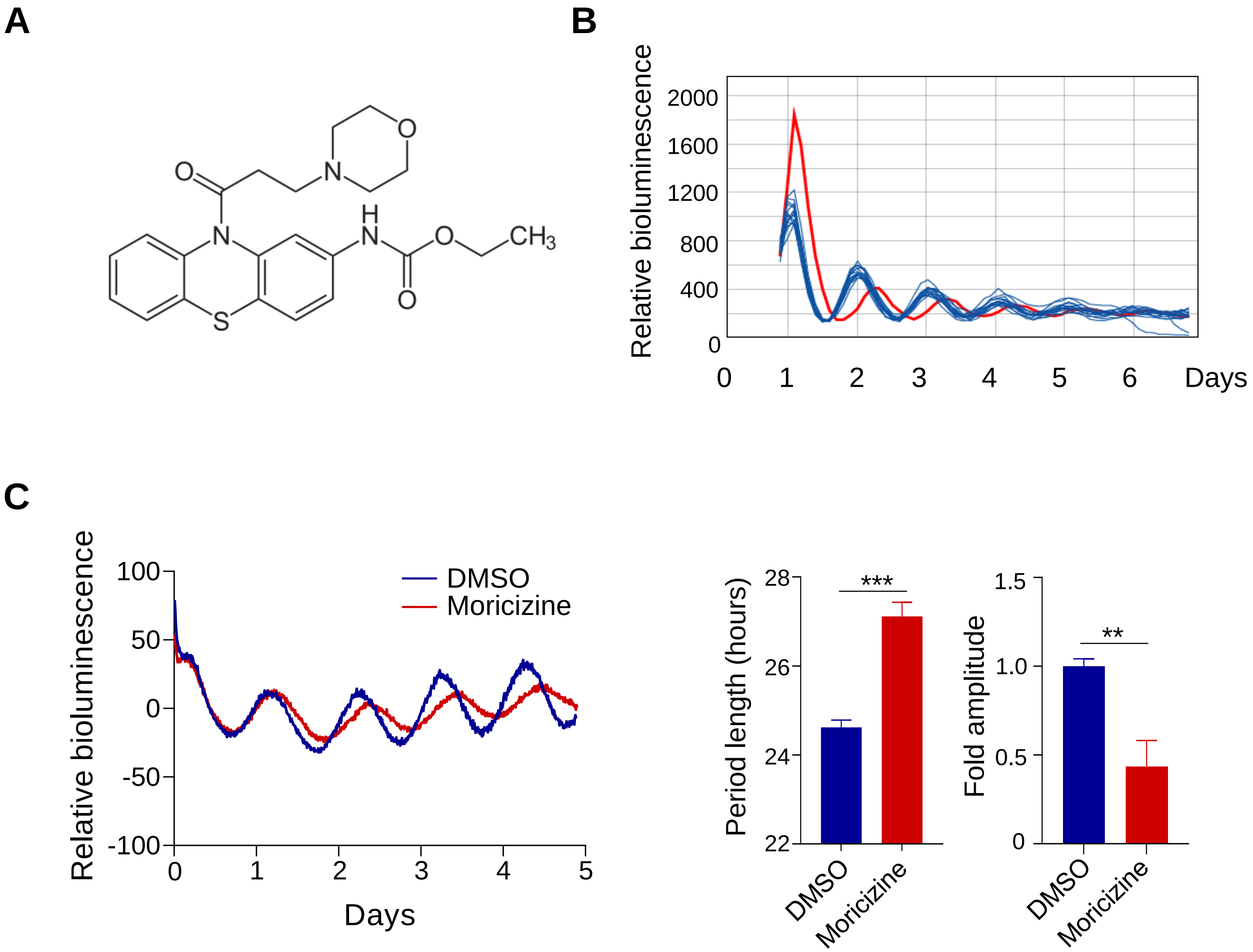

Figure S1. (**A**) Chemical structure of moricizine. (**B**) Initial high-throughput screening result in a 384-well plate. Reporter rhythms in Per2::LucSV cells pretreated with 5 µM Fsk from the initial primary screen. Blue traces: DMSO. red: moricizine, 5 µM. **(C)** Representative PER2::LUC bioluminescence recording, average circadian period lengths and amplitudes of Per2::LucSV fibroblast cells pretreated with 5 µM Fsk followed by DMSO or moricizine (10 mM) treatment (n=3-4 for each treatment). Data are presented as mean ± SEM. T-test shows the significant statistical differences between DMSO and moricizine. (**, p<0.01; ***, p<0.001).

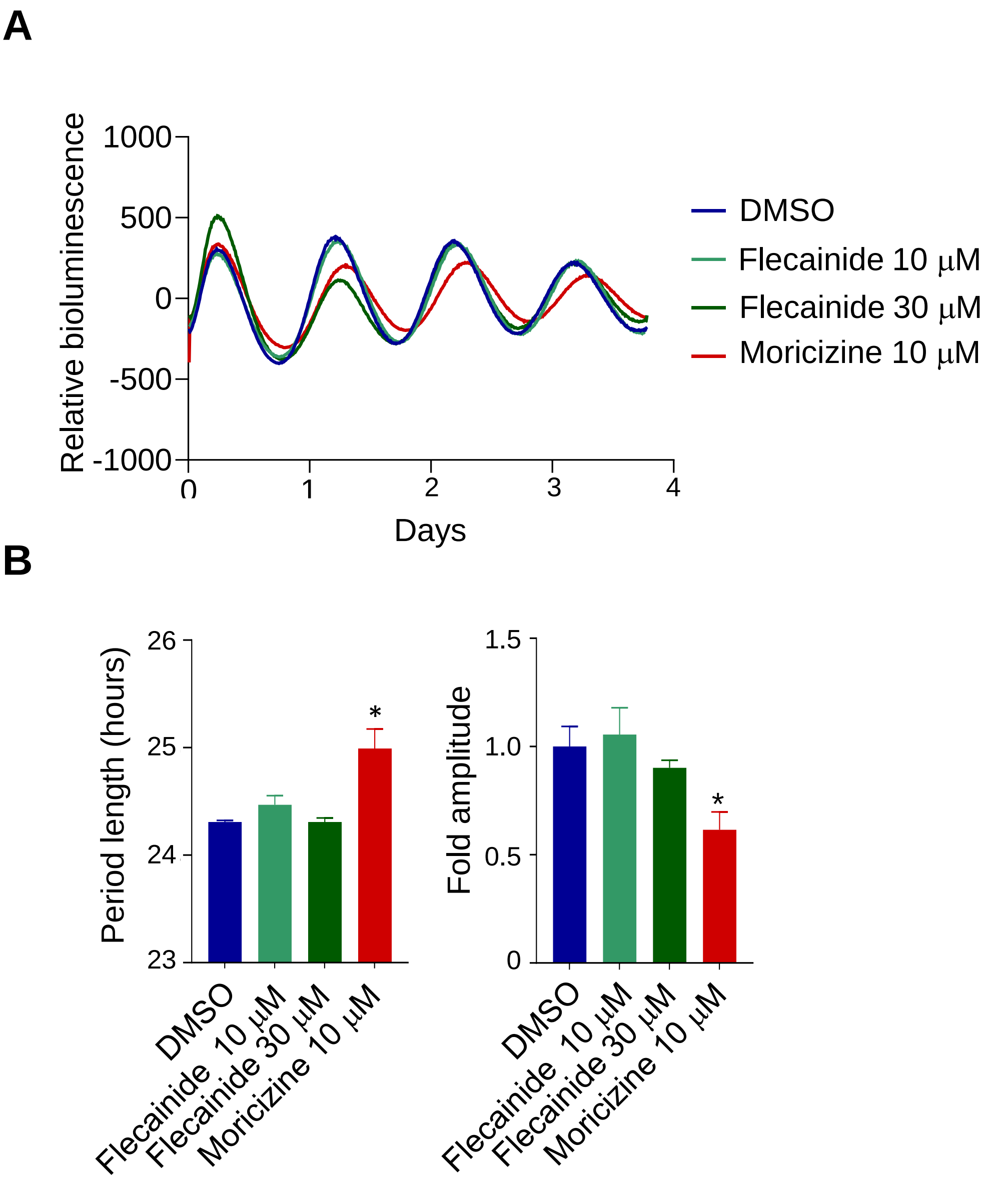

Figure S2. (A) Representative PER2::LUC bioluminescence recording of Per2::LucSV fibroblast cells treated with DMSO, flecainide (10 μM or 30 μM) or moricizine (10 μM). (B) Average circadian period lengths and amplitudes are shown (n=3-4). Data are presented as mean±SEM. T-test shows significant statistical differences between DMSO and flecainide or moricizine. (*, $p<0.05$).

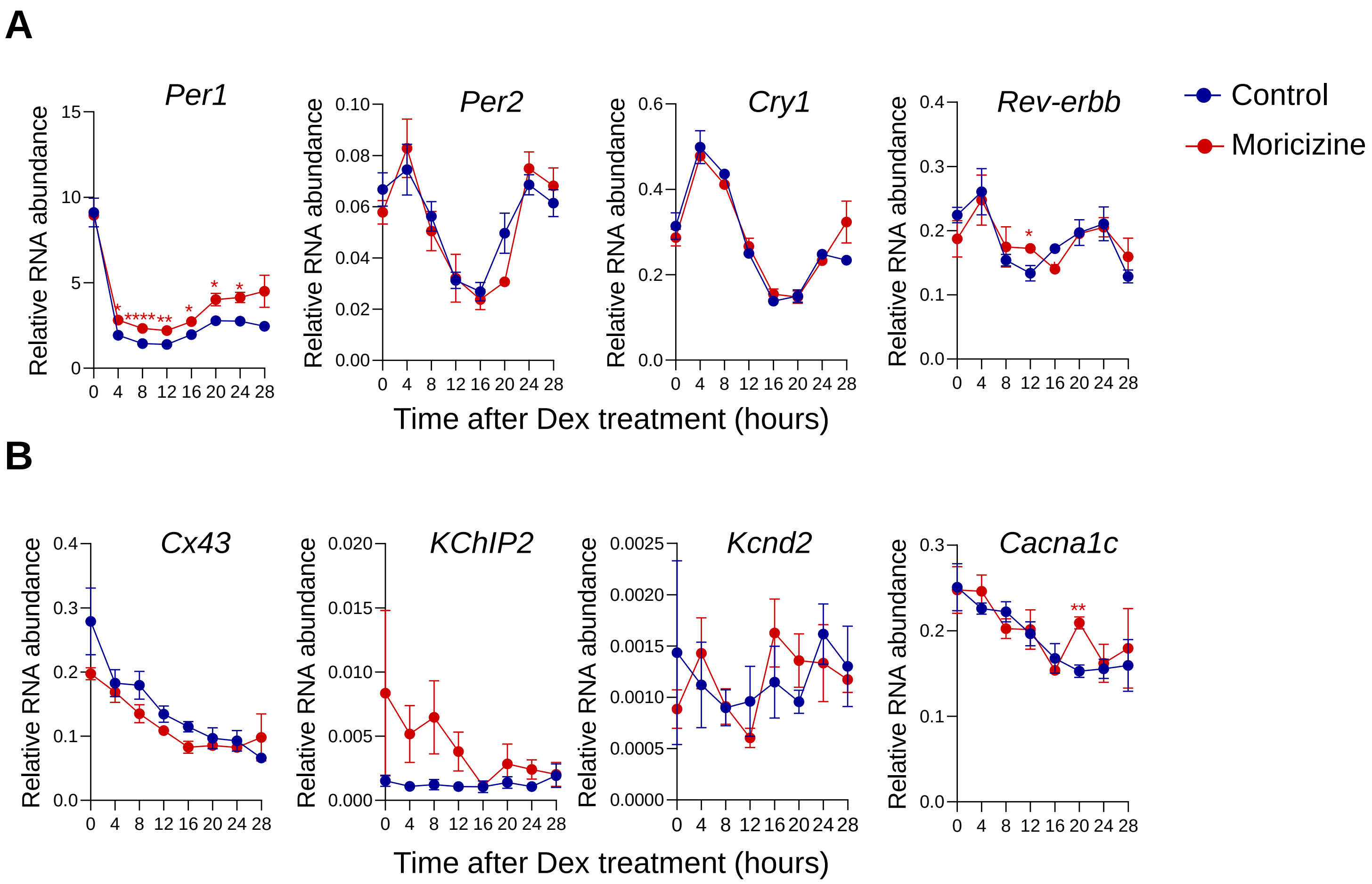

Figure S3. Real-time qPCR analysis in reporter cells. Per2::lucSV reporter cells were treated with vehicle control (blue) and moricizine 20 μM (red). (**A**) Circadian gene expression (*Per1*, *Per2, Cry1, and Rev-erbb*). (**B**) Cardiac channel gene expression (*Cx43, KChIP2, Kcnd2,* and *Cacna1c*). Data are shown as mean± SEM every 4 hrs for 28 hrs (n=3). T-test shows the significant statistical differences between DMSO and moricizine. (*, $p<0.05$; **, $p<0.01$; ***, $p<0.001$).

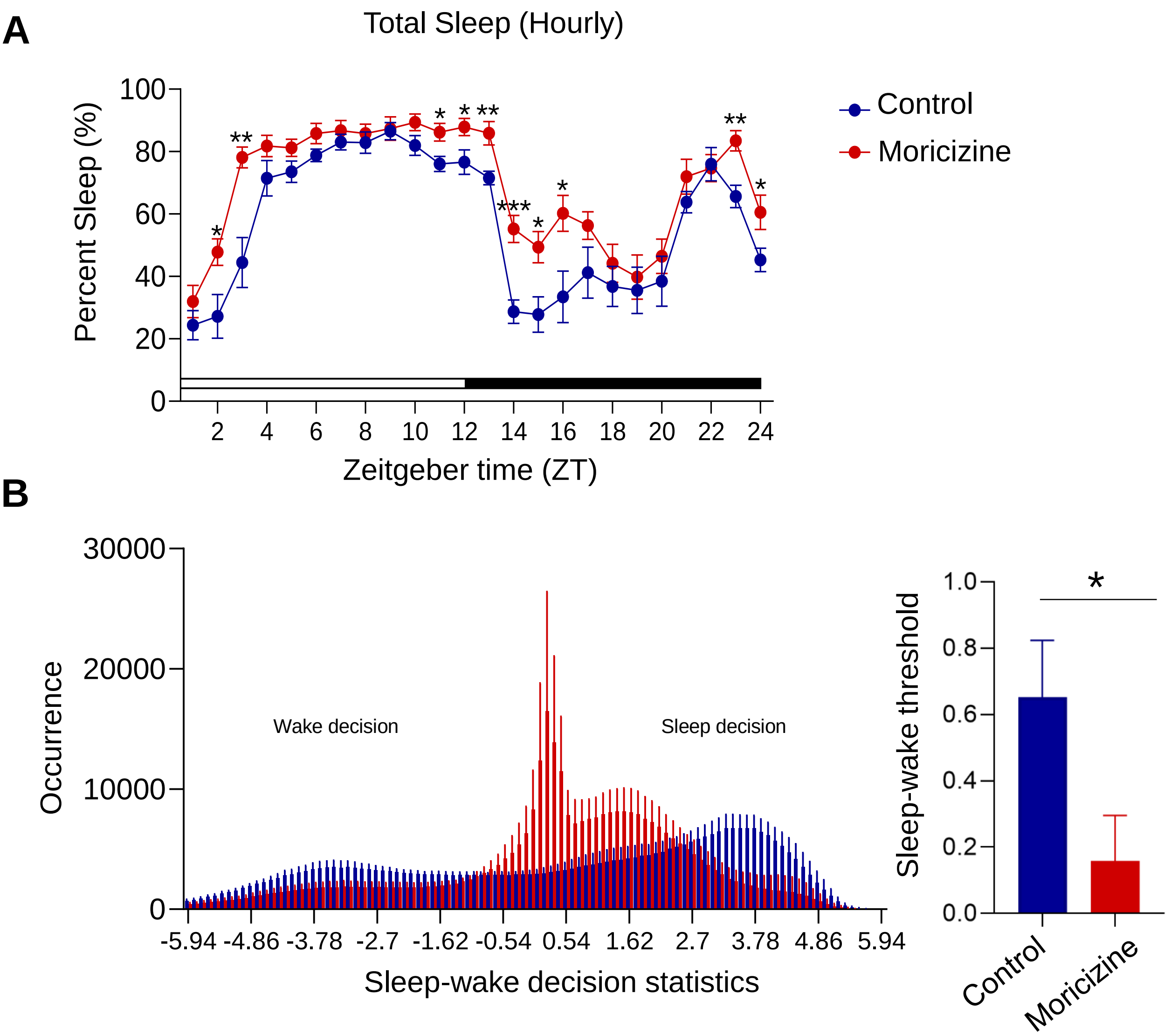

Figure S4. Moricizine alters sleep in C57BL6/J mice. (**A**) Hourly percentage of sleep is shown over the 12:12 light:dark cycle (LD) as indicated by the white and black bars (**B**) Left: Sleep-wake decision statistics histogram and threshold in Vehicle (PBS, blue, n=8) and Moricizine (1.25 mg per mouse, red, n=8). Right: The decision threshold is computed by using the SleepStats software to account for variations in mouse behavior and weight based on the clustering of decision statistics. Decision statistics above the threshold are classified as sleep. Data are presented as mean ± SEM. T-test shows the significant statistical differences between control and moricizine treated group. (*, $p<0.05$; **, $p<0.01$; ***, $p<0.001$).

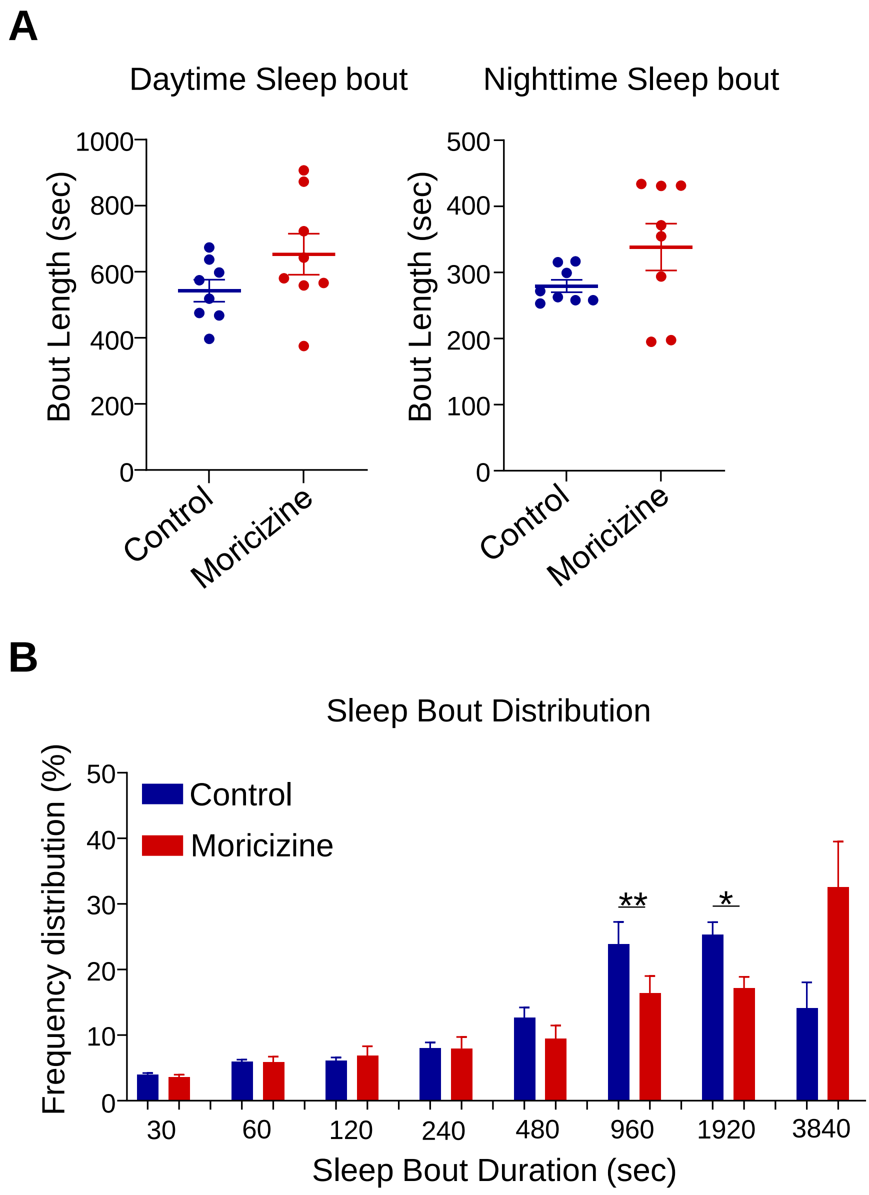

Figure S5. Moricizine effects on sleep quality in C57BL6/J mice. (**A**) Daytime sleep bout length and nighttime sleep bout length. (**B**) Sleep bout length distribution of mice with vehicle (PBS, blue, n=8) and moricizine (1.25 mg per mouse, red, n=8). Data are presented as mean ± SEM. T-test shows the significant statistical differences between control and moricizine treated group. (*, $p<0.05$; **, $p<0.01$).

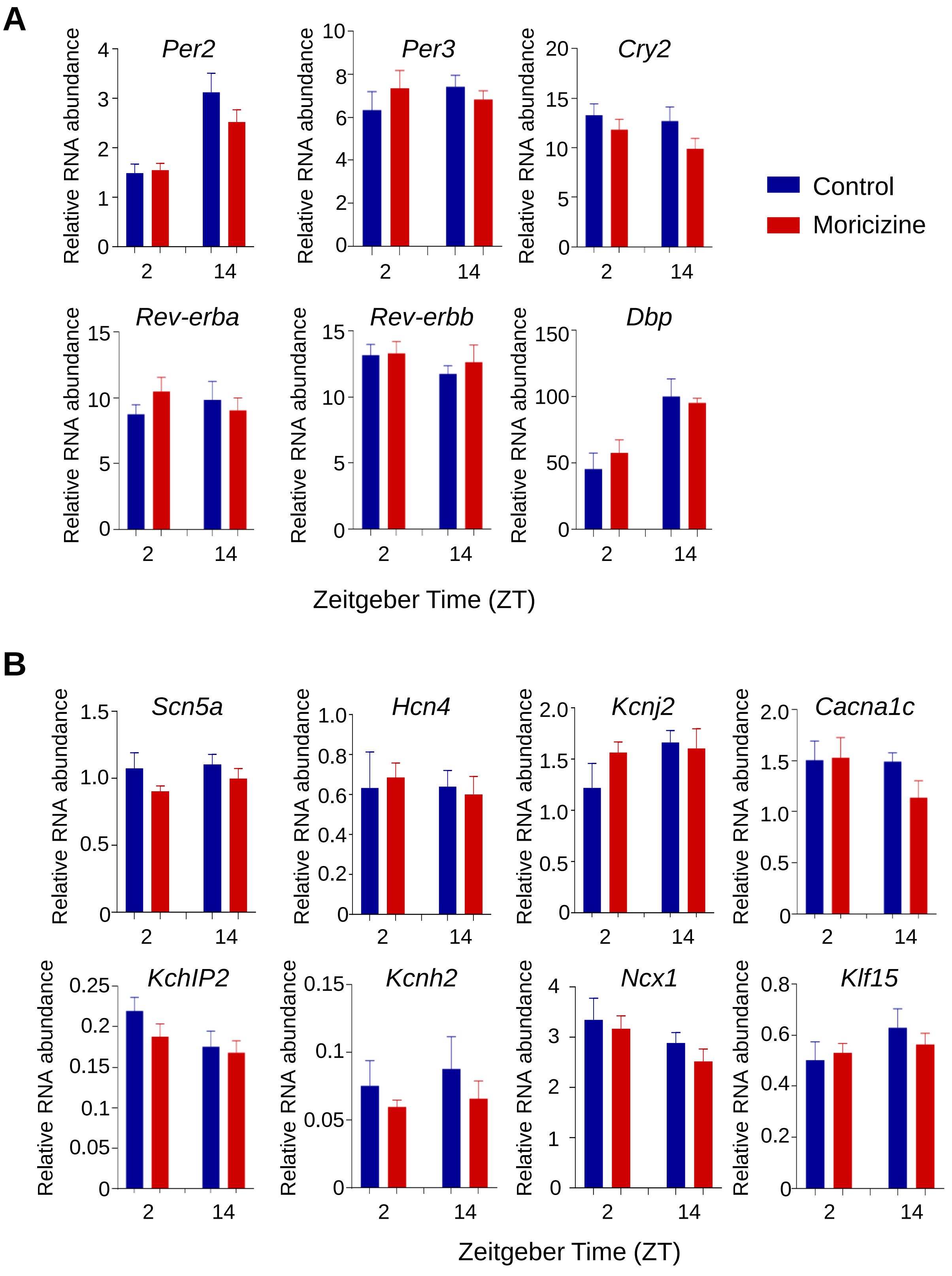

Figure S6. Real-time qPCR analysis in heart tissues. Hearts were collected from control (blue bar, n=4) and moricizine (red bar, n=4) treated mice at ZT2 and ZT14 and analyzed for circadian gene expression. (**A**) *Per2, Per3, Cry2, Rev-erba, Rev-erbb,* and *Dbp*) and cardiac (**B**) *Scn5a, Hcn4, Kcnj2, Cacna1c, KchIP2, Kcnh2, Ncx1,* and *Klf15*) gene expression. Data are presented as mean± SEM. T-test shows the significant statistical differences between control and moricizine treated group.

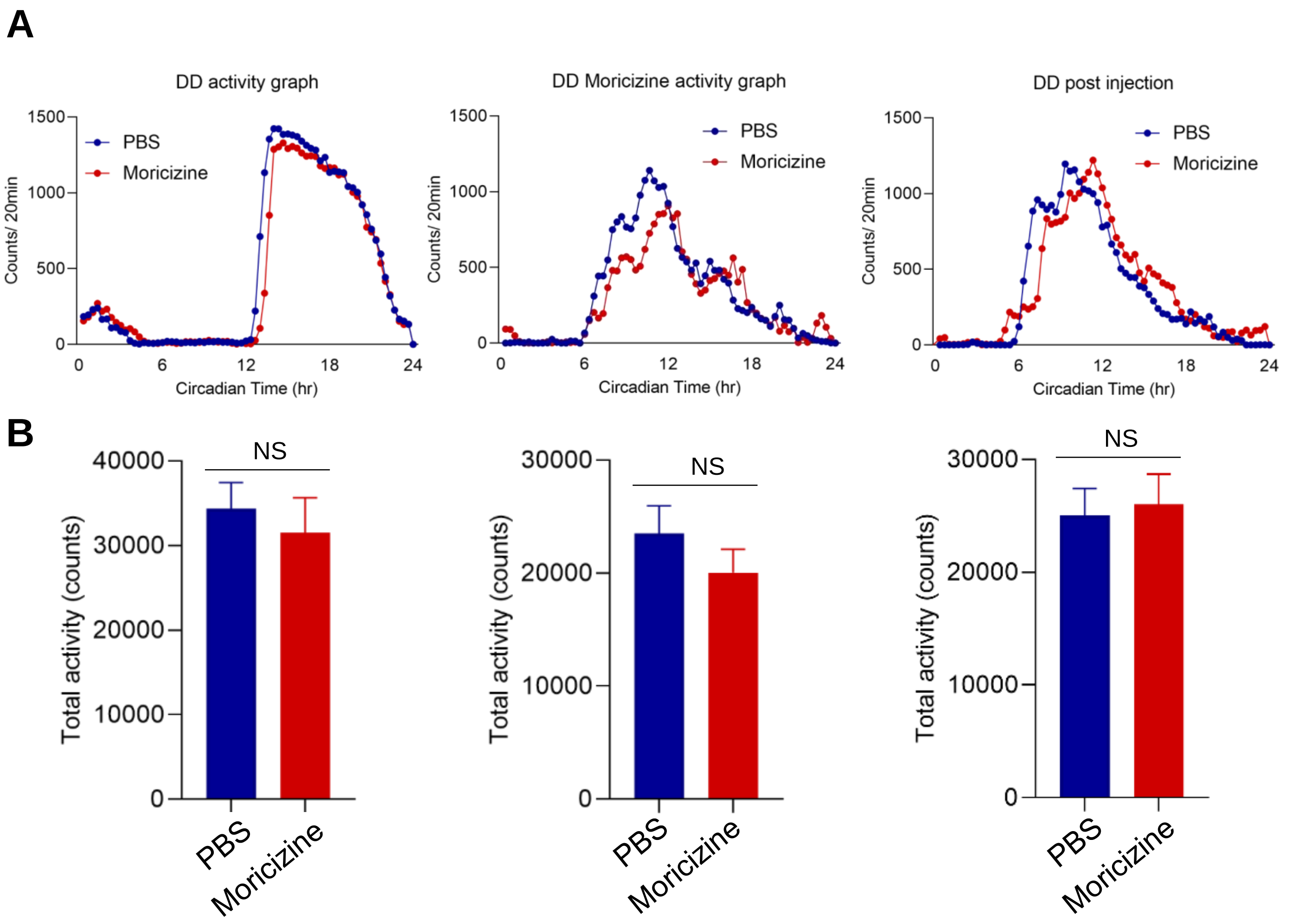

Figure S7. Effects of Moricizine on wheel-running activity in C57BL/6J mice. (**A**) Average wave plots summarizing wheel-running activity during DD for before PBS (blue, N=7) or moricizine (red, N=6) injection, during injection, and post injection. (**B**) Daily total wheel-running activity during DD for before injection, during injection, and post injection.
